# Characterization of the complete chloroplast genome of *Gerbera jamesonii* Bolus in China and phylogenetic relationships

**DOI:** 10.1080/23802359.2019.1644230

**Published:** 2019-07-23

**Authors:** Yong-Yan Zhang, Fan Liu, Xing-qi Wang, Xiao-bao Shi, Na Tian, Zhong-Xiong Lai, Chun-Zhen Cheng

**Affiliations:** College of Horticulture/Institute of Horticultural Biotechnology, Fujian Agriculture and Forestry University, Fuzhou, P. R. China

**Keywords:** *Gerbera jamesonii* Bolus, chloroplast genome, Compositae, phylogenetic relationship analysis

## Abstract

Gerbera (*Gerbera jamesonii* Bolus) is one of the most popular ornamental flowers and one of the top five cut flowers worldwide. In this study, we presented the complete chloroplast genome of it using BGISEQ-500 sequencing. Its chloroplast genome is 151,898 bp in size, containing a large single copy region (83,518 bp) and a small single copy region (18,244 bp), and a pair of IR regions (25,068 bp). The chloroplast genome contains 113 unique genes, including 80 protein-coding genes, 29 tRNAs, and 4 rRNAs. Phylogenetic maximum likelihood analysis based on chloroplast genomes of Gerbera and other 17 plant species indicated that the relationship between Gerbera and other plant species of family Compositae is not very close and its relationship with *Atractylodes lancea* is the closest.

Gerbera (*Gerbera jamesonii* Bolus), also known as Transvaal or Barbeton daisy, belongs to family Compositae and is native to South Africa and Asia (Minerva and Kumar 2012). It is one of the most popular ornamental flowers worldwide (Parthasarathy and Nagaraju 1999) and is one of the great commercial importance with multiple applications such as cut flower, pot flower, and garden plants (Hansen 1999). Many chloroplast genomes of plant species from family Compositae have been reported. However, up to now, people’s understanding of the chloroplast genome of Gerbera is very limited. In this study, in order to provide information about the chloroplast genome and to facilitate the further genetic studies of Gerbera, we assembled and characterized the complete chloroplast genome using BGISEQ-500 sequencing data. In addition, we explored the phylogenetic relationship of Gerbera with other plant species, which can be subsequently used for valuable species researches of Compositae.

The specimen sample of *G. jamesonii* was collected from test field of Institute of Horticultural Biotechnology, Fujian Agriculture and Forestry University in Fuzhou (Fujian, China, 26°05′20″N; 119°13′46″E) and samples were deposited at Fujian Agriculture and Forestry University. The total genomic was extracted from fresh leaves using Plant Genomic DNA Kit (Tiangen Biotech, Beijing, China) and stored at the Fujian Agriculture and Forestry University (No. G03). By using BGISEQ-500 sequencing, approximately 1.2 Gbp high-quality reads were obtained and subjected to sequence alignment with chloroplast genomes of 14 plant species from Compositae, including: *Xanthium sibiricum* (NC_042232.1), *Centaurea diffusa* (NC_024286.1), *Silybum marianum* (KT267161.1), *Guizotia abyssinica* (EU549769.1), *Jacobaea vulgaris* (NC_015543.1), *Cynara humilis* (NC_027113.1), *Jacobaea vulgaris* (HQ234669.1), *Cynara humilis* (KP299292.1), *Artemisia montana* (NC_025910.1), *Sonchus canariensis* (MK033506.1), *Sonchus acaulis* (MK033507.1), *Sonchus webbii* (MK033508.1), *Lactuca sativa* (AP007232.1) and *Artemisia frigida* (NC_020607.1). Then, reads were assembled into contigs using CLC Genomics Workbench v8.0 (CLC Bio, Aarhus, Denmark). DOGMA (Wyman et al. 2004) and Geneious (Kearse et al. 2012) were then used for the annotation of the assembled chloroplast genome, which has been deposited in Genbank with the accession number MN087227.

The complete chloroplast genome of *G. jamesonii* is 151,898 bp in size, containing a large single copy region (83,518 bp) and a small single copy region (18,244 bp), and a pair of IR regions (25,068 bp). The chloroplast genome contains 113 unique genes, including 80 protein-coding genes, 29 tRNAs, and 4 rRNAs. In the IR regions, a total of 18 genes, including seven protein-coding genes (i.e. *ycf2*, *ycf15*, *rps12*, *rps7*, *rpl23*, *rpl2,* and *ndhB*), 7 tRNAs (i.e. *trnV-GAC*, *trnR-ACG*, *trnN-GUU*, *trnL-CAA*, *trnI-GAU*, *trnI-CAU*, and *trnA-UGC*), and 4 rRNAs (*4.5S*, *5S*, *16S,* and *23S* rRNAs) were found duplicated. The overall nucleotide composition of the chloroplast genome is 30.9% A, 31.3% T, 18.5% C, and 19.2% G with the total GC content of 37.7%.

For phylogenetic maximum likelihood analysis, we downloaded the chloroplast genomes of 15 plant species belonging to Compositae and 2 plant species from Campanulaceae (as outgroup) from GenBank to access the relationship of Gerbera with them. HomBlocks pipeline (Bi et al. 2018) and RAxML-HPC2 on XSEDE version 8.2.10 (Stamatakis 2014) were respectively used for chloroplast genome sequence alignment and construction of maximum likelihood (ML) tree. 1000 bootstrap replicates were used to calculate the bootstrap values of the ML tree. The result showed that the relationship between Gerbera and other plant species of family Compositae is not very close and its relationship with *Atractylodes lancea* from Cynareae is the closest (Figure 1).

**Figure 1.**
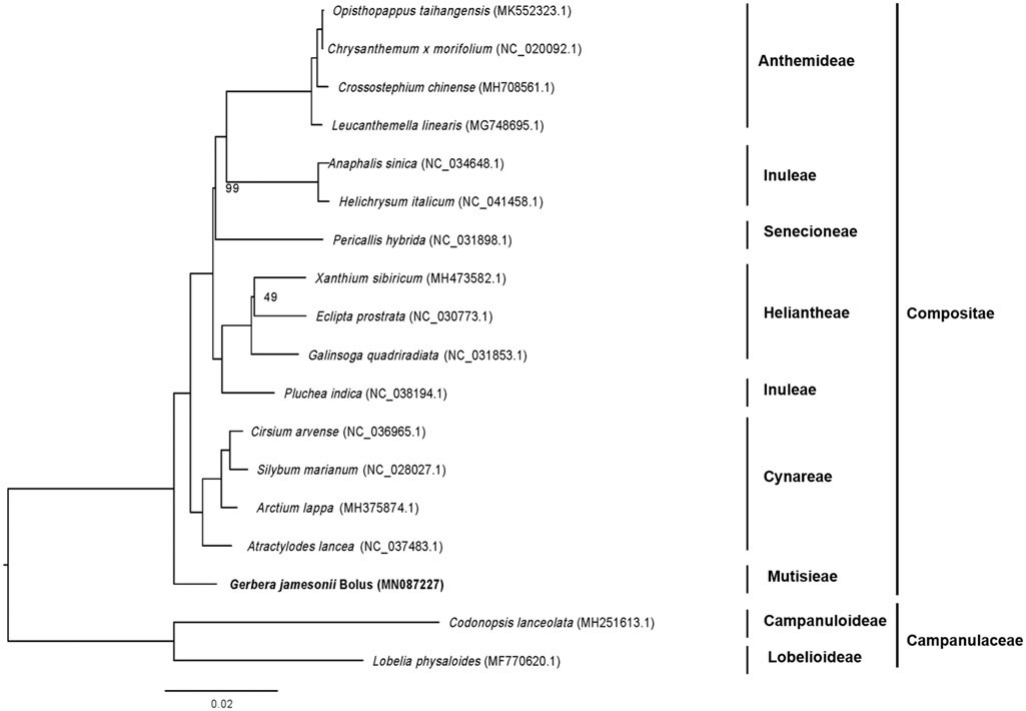
Maximum likelihood phylogenetic tree based on the complete chloroplast genome sequences of 18 plant species, with *Codonopsis lanceolata* and *Lobelis physalodies* as outgroup. Numbers on the nodes are bootstrap values with 1000 replicates and bootstrap values of 100 were omitted.
